# Verification of Varian enhanced dynamic wedge implementation in MasterPlan treatment planning system

**DOI:** 10.1120/jacmp.v10i2.2867

**Published:** 2009-04-22

**Authors:** Massimo Pasquino, Valeria Casanova Borca, Santi Tofani, F. Ozzello

**Affiliations:** ^1^ S.C. Fisica Sanitaria Azienda Sanitaria ASL TO4 Ivrea (TO) Italy; ^2^ S.C. Radioterapia Azienda Sanitaria ASL TO4 Ivrea (TO) Italy

**Keywords:** radiotherapy, enhanced dynamic wedge, treatment planning

## Abstract

This paper investigates the accuracy of the two available calculation algorithms of the Oncentra MasterPlan three‐dimensional treatment planning system (TPS) – the pencil beam method and collapsed‐cone convolution – in modeling the Varian enhanced dynamic wedge (EDW). Measurements were carried out for a dual high energy (6–15 MV) Varian DHX‐S linear accelerator using ionization chambers for beam axis measurements (wedge factors and depth doses), film dosimetry for off‐axis dose profiles measurements, and a diode matrix detector for two dimensional absolute dose distributions. Using both calculation algorithms, different configuration of symmetric and asymmetric fields varying the wedge's angle were tested. Accuracy of the treatment planning system was evaluated in terms of percentage differences between measured and calculated values for wedge factors, depth doses, and profiles. As far as the absolute dose distribution was concerned, the gamma index method (Low et al.^(13)^) was used with 3% and 3 mm as acceptance criteria for dose difference and distance‐to‐agreement, respectively. Wedge factors and percentage depth doses were within 1% deviation between calculated and measured values. The comparison of measured and calculated dose profiles shows that the Van Dyk's acceptance criteria (Van Dyk et al.^(14)^) are generally met; a disagreement can be noted for large wedge angles and field size limited to the low dose‐low gradient region only. The 2D absolute dose distribution analysis confirms the good accuracy of the two calculation algorithms in modeling the enhanced dynamic wedge.

PACS number: 87.53.Bn, 87.55.kn, 87.56.ng

## I. INTRODUCTION

The enhanced dynamic wedge (EDW) of the Varian linacs consists in the simulation of a physical wedge by moving one of the Y collimator jaws during the irradiation with variable speed from its maximum open position to 0.5 cm of the opposite jaw and adjusting dose rate during treatment.

The wedged dose distributions are generated by means of a single golden segmented treatment table (SGTT) that is specifically for the 60° wedge angle of each energy, and is used to control the position of the moving jaw versus the percentage of delivered monitor units. Then, combining open and 60° wedged beams, other wedge angles (10°, 15°, 20°, 25°, 30° and 45° degrees) can be reproduced.

The dynamic wedges overcome unfavorable characteristics of the physical wedges (beam hardening, radiation therapist involvement). They also provide some advantages either in clinical practice (greater dose homogeneity^(1)^ within the target area and reduction of peripheral dose^(2)^), or in radioprotection by reducing the dose to the staff member operating the linac and thereby avoiding neutron activation reactions which can be produced in high energy photon beam using physical wedges.^(3)^

Clinical implementation of EDW requires the verification of treatment planning system (TPS) capability to reproduce this type of modulation of the radiation field intensity. This is achieved by comparing measured and calculated data (EDW factors, PDD, dose profiles, and dose maps) as has been studied by several authors for different TPSs.

Weber et al.^(4)^ in 1996 and Chelminski et al.^(5)^ in 2006 analyzed Helax TMS TPS, comparing calculated depth doses, profiles and output factors in dynamic wedge fields with the measured ones. In 1999, Papatheodorou et al.^(6)^ verified output factors and dose profiles calculations performed by ISiS3D TPS by measurements. A year later, Miften et al.^(7)^ commissioned Varian's EDW in Focus TPS, comparing the computed dose distribution and wedge factors against the measured ones. In 2004 and 2005 respectively, Shao et al.^(8)^ and Alaei et al.^(9)^ studied the accuracy of EDW calculation of ADAC Pinnacle RTP, comparing measured and calculated dose profiles and output factors. Finally, in 2007, Caprile et al.^(10)^ used the two calculation algorithms of the Varian Eclipse TPS to compare measured and calculated two‐dimensional EDW dose distributions.

The main purpose of the present paper is to verify the implementation of the Varian EDW into the Oncentra MasterPlan TPS by comparing calculated EDW factors, depth doses, profiles and two‐dimensional dose distributions with measured ones, and to compare the obtained results with those published for different treatment planning systems.^(^ ^4^ ^–^ ^10^ ^)^

## II. MATERIALS AND METHODS

A dual high‐energy (6–15 MV) linear accelerator, DHX‐S (Varian Medical Systems, Inc., Palo Alto, CA), installed at the Radiotherapy Department of Ivrea Hospital, was used to generate wedge‐shaped dose distributions by means of the EDW.

The dose distributions were calculated by the Oncentra MasterPlan (Nucletron B.V., Veenendaal, the Netherlands) version 3.0 TPS, whose beam modeling supports two different dose calculation methods: one based on the superposition of energy deposition kernels of pencil beams (PB),^(11)^ and one based on the collapsed cone (CC) superposition of points kernels.^(12)^

Oncentra MasterPlan supports dose calculation of dynamic collimation obtained with Varian enhanced dynamic wedge technique. The modulation induced at a point by a dynamically modulated field is, to the first order, proportional to the radiation delivered during those intervals when the point is directly exposed to the radiation source. Second order phenomena such as leakage through the collimators, scattering from the treatment head and the perturbation of the monitor signal from collimator backscatter, are also included.

The treatment unit characterization process is executed by the system vendor and determines the beam modeling parameters (head scatter parameters, kernels, beam source size, attenuation coefficient, fluence distribution), starting from measurements of a specified set of data consisting of depth doses, off‐axis profiles, output factors and head scatter factors for open static beams. The implementation of EDW into the TPS does not require additional measurements: the only additional input data is the Varian golden STT used in conjunction with the input data measured in air and water for open beams.^(4)^ Moreover, no adjustable parameters are available for the customer that would improve the agreement between calculated and measured data.

We evaluated the TPS accuracy by comparing measurements and calculations performed under the same conditions, based on a phantom created by the TPS with a homogeneous density of $1\;g/{\text{cm}}^{3}$. The slice spacing of the phantom was 2 mm and the calculation grid was 2×2 mm for the PB and CC calculation algorithm.

Measurements were performed using the $Y_{1}$‐IN wedge orientation and the reference geometrical configuration suggested by Nucletron in performing the measurements requested to simulate the treatment machine (SSD 90 cm and 10 cm depth).

Beam axis measurements (EDW factors and depth doses) were performed in a water phantom Blue Phantom (Scanditronix Wellhofer, Uppsala, Sweden) using an IC 13 ionization chamber (Scanditronix Wellhofer, Uppsala, Sweden) positioned at the beam central axis perpendicular to the wedge direction.

EDR2 Ready‐Pack films (Carestream Health, Rochester, NY) were used for relative off‐axis dose distribution measurements. The film calibration obtained at a specific depth from the static open field was applied to the EDW fields, because the beam spectrum does not vary significantly from the EDW field to the open field. To reduce the variability, film calibration and dosimetry measurements were performed in the same session.

Films were irradiated at 10 cm depth in the water equivalent phantom T29672 (PTW, Freiburg). The irradiated flms were then digitized with a laser scanning photodensitometer VXR‐12 Plus (Vidar Systems Corporation, Herndon, VA) having a light spot size of 85 μm diameter and a spatial resolution of approximately 300 dpi. Measured and calculated profiles were normalized at the collimator axis and analyzed using the RIT113 software (ver.4.4, Radiological Imaging Technology, Inc., Colorado), that supplies the operator with different tools including isodose overlay, dose difference (DD) and distance‐to‐agreement (DTA) plot and histograms and gamma evaluation method.^(13)^ Measured and calculated dose profiles were compared using the regions suggested by the Van Dyk's analysis:^(14)^ high dose–high gradient areas (HDHG, >30%/cm), high dose–low gradient areas (HDLG, 80% of field size), and low dose–low gradient areas (LDLG, <7% of normalization dose). Moreover, the algorithms' behaviour in the toe of the wedged profiles was studied.

Finally, absolute 2D dose distributions were measured by means of MapCHECK Model 1175 (Sun Nuclear Corporation, Melbourne, FL) diode matrix detector, which can perform absolute and relative measurements, making it a useful instrument in performing dose measurements in intensity modulated fields.^(15)^ The MapCHECK consists of 445 n‐type diode detectors arranged in a 22 cm octagonal grid having two detector densities: the $10\times 10\;{\text{cm}}^{2}$ central area that contains 221 diodes spaced 10 mm arranged on lines spaced 5 mm; the remaining 224 diodes are arranged 20 mm in lines spaced 10 mm in the outer area. Detectors have an inherent buildup of $2\;g/{\text{cm}}^{2}$ and an active area of $0.8\times 0.8\;{\text{mm}}^{2}$; they lie 1.35 cm under the top with an inherent backscatter thickness of $2.27\;g/{\text{cm}}^{2}$. The absolute dose calibration was performed using a $10\times 10\;{\text{cm}}^{2}$ open field positioning the diode matrix at the isocenter with a 10 cm thickness of the PTW water equivalent phantom, and relating the reading of the central axis detector with the dose measured in the same condition with a Farmer‐type ionization chamber M30001 (PTW, Freiburg).

The MapCHECK measures the integrated absolute dose at all detector locations, correcting each reading to the central detector sensitivity. Then this measured dose map can be compared to an imported treatment plan dose map using the gamma index method with user defined acceptance criteria for DD and DTA, and calculating the percentage of examined points resulting in γ values lower or equal to 1 (the so‐called passing rate). Points that lie outside the defined tolerance levels are directly painted on the calculated dose map. Therefore, measured and calculated dose distribution can be overlayed and dose profiles can be extracted from any X and Y detector line and compare with the calculated ones.

Following are the beam axis and off‐axis measurement protocols we considered.

### A. Beam Axis

#### A.1. EDW Factors

EDW factor is defined as the ratio between the ion chamber integrated reading on the central axis of a wedged field and the integrated reading at the same depth for the open field having the same size and for the same number of monitor units.

The EDW factors were measured at 10 cm depth for symmetric fields ranging from $5\times 5\;{\text{cm}}^{2}$ to $20\times 20\;{\text{cm}}^{2}$ and for some fields asymmetric on Y jaws only, as illustrated in Tables 1 and 2.

**Table 1:** 6 MV EDW factor measured values vs. calculated ones obtained by using Pencil Beam (PB) and Collapsed Cone (CC) algorithm.

*Field*	*Wedge Angle*	$WF_{mea}$	$WF_{PB}$	*%diff*	$WF_{CC}$	*%diff*
$X=5\;\text{cm},\;Y_{1}=1\;\text{cm},Y_{2}=4\;\text{cm}$	15°	0.940	0.945	0.5	0.945	0.5
	30°	0.893	0.890	‐0.3	0.890	‐0.3
	60°	0.735	0.731	‐0.6	0.732	‐0.5
$X=5\;\text{cm},\;Y_{1}=2.5\;\text{cm},Y_{2}=2.5\;\text{cm}$	10°	0.981	0.978	‐0.3	0.980	‐0.1
	15°	0.972	0.969	‐0.3	0.969	‐0.3
	20°	0.961	0.958	‐0.3	0.959	‐0.2
	25°	0.952	0.947	‐0.5	0.948	‐0.3
	30°	0.940	0.936	‐0.4	0.936	‐0.5
	45°	0.901	0.894	‐0.7	0.894	‐0.8
	60°	0.840	0.829	‐1.3	0.829	‐1.3
$X=5\;\text{cm},\;Y_{1}=4\;\text{cm},Y_{2}=1\;\text{cm}$	15°	0.996	0.991	‐0.5	0.990	‐0.6
	30°	0.982	0.980	‐0.2	0.979	‐0.3
	60°	0.946	0.940	‐0.6	0.940	‐0.7
$X=10\;\text{cm},\;Y_{1}=2\;\text{cm},Y_{2}=8\;\text{cm}$	15°	0.869	0.870	0.1	0.872	0.3
	30°	0.754	0.758	0.5	0.756	0.2
	60°	0.513	0.517	0.7	0.515	0.3
$X=10\;\text{cm},\;Y_{1}=5\;\text{cm},Y_{2}=5\;\text{cm}$	10°	0.951	0.950	0.0	0.949	‐0.2
	15°	0.927	0.927	0.0	0.925	‐0.2
	20°	0.904	0.902	‐0.2	0.902	‐0.2
	25°	0.880	0.879	‐0.1	0.877	‐0.3
	30°	0.856	0.854	‐0.2	0.852	‐0.4
	45°	0.774	0.770	‐0.6	0.769	‐0.6
	60°	0.666	0.659	‐1.1	0.658	‐1.2
$X=10\;\text{cm},\;Y_{1}=8\;\text{cm},Y_{2}=2\;\text{cm}$	15°	0.971	0.975	0.4	0.975	0.4
	30°	0.944	0.945	0.2	0.945	0.1
	60°	0.847	0.847	0.0	0.849	0.3
$X=15\;\text{cm},\;Y_{1}=7.5\;\text{cm},Y_{2}=7.5\;\text{cm}$	10°	0.917	0.917	0.0	0.916	‐0.1
	15°	0.879	0.879	0.0	0.877	‐0.2
	20°	0.843	0.842	‐0.2	0.840	‐0.4
	25°	0.807	0.806	‐0.1	0.804	‐0.4
	30°	0.772	0.770	‐0.2	0.769	‐0.4
	45°	0.661	0.660	‐0.2	0.658	‐0.5
	60°	0.533	0.528	‐0.9	0.527	‐1.1
$X=20\;\text{cm},\;Y_{1}=10\;\text{cm},Y_{2}=10\;\text{cm}$	10°	0.877	0.879	0.2	0.876	‐0.2
	15°	0.824	0.827	0.3	0.823	‐0.1
	20°	0.776	0.777	0.0	0.773	‐0.4
	25°	0.730	0.731	0.1	0.728	‐0.2
	30°	0.686	0.687	0.1	0.684	‐0.3
	45°	0.560	0.559	‐0.2	0.557	‐0.6
	60°	0.428	0.424	‐0.7	0.423	‐1.1

**Table 2:** 15 MV EDW factor measured values vs. calculated ones obtained by using Pencil Beam (PB) and Collapsed Cone (CC) algorithm.

*Field*	*Wedge Angle*	$WF_{mea}$	$WF_{PB}$	*%diff*	$WF_{CC}$	*%diff*
$X=5\;\text{cm},\;Y_{1}=1\;\text{cm},Y_{2}=4\;\text{cm}$	15°	0.951	0.957	0.6	0.957	0.6
	30°	0.920	0.911	‐0.9	0.911	‐0.9
	60°	0.783	0.774	‐1.1	0.775	‐0.9
$X=5\;\text{cm},\;Y_{1}=2.5\;\text{cm},Y_{2}=2.5\;\text{cm}$	10°	0.984	0.984	0.0	0.984	0.0
	15°	0.976	0.976	‐0.1	0.977	0.0
	20°	0.968	0.967	0.0	0.967	‐0.1
	25°	0.959	0.958	‐0.1	0.958	‐0.1
	30°	0.949	0.949	‐0.1	0.949	‐0.1
	45°	0.916	0.915	‐0.1	0.914	‐0.2
	60°	0.862	0.860	‐0.2	0.859	‐0.4
$X=5\;\text{cm},\;Y_{1}=4\;\text{cm},Y_{2}=1\;\text{cm}$	15°	0.995	0.994	‐0.1	0.993	‐0.2
	30°	0.985	0.986	0.1	0.984	‐0.1
	60°	0.956	0.956	0.0	0.955	‐0.1
$X=10\;\text{cm},\;Y_{1}=2\;\text{cm},Y_{2}=8\;\text{cm}$	15°	0.899	0.901	0.2	0.900	0.1
	30°	0.804	0.807	0.4	0.806	0.2
	60°	0.583	0.585	0.3	0.582	‐0.2
$X=10\;\text{cm},\;Y_{1}=5\;\text{cm},Y_{2}=5\;\text{cm}$	10°	0.961	0.963	0.3	0.960	‐0.1
	15°	0.942	0.945	0.3	0.942	0.0
	20°	0.923	0.926	0.3	0.922	‐0.1
	25°	0.903	0.905	0.2	0.902	‐0.1
	30°	0.883	0.885	0.2	0.881	‐0.2
	45°	0.813	0.814	0.2	0.811	‐0.2
	60°	0.715	0.716	0.2	0.713	‐0.3
$X=10\;\text{cm},\;Y_{1}=8\;\text{cm},Y_{2}=2\;\text{cm}$	15°	0.981	0.981	0.0	0.981	0.0
	30°	0.957	0.960	0.3	0.958	0.1
	60°	0.883	0.882	‐0.1	0.883	0.0
$X=15\;\text{cm},\;Y_{1}=7.5\;\text{cm},Y_{2}=7.5\;\text{cm}$	10°	0.933	0.939	0.6	0.935	0.1
	15°	0.904	0.909	0.6	0.904	0.1
	20°	0.874	0.879	0.6	0.874	0.0
	25°	0.846	0.849	0.4	0.844	‐0.2
	30°	0.815	0.819	0.6	0.814	‐0.1
	45°	0.720	0.722	0.3	0.717	‐0.3
	60°	0.596	0.599	0.6	0.595	‐0.2
$X=20\;\text{cm},\;Y_{1}=10\;\text{cm},Y_{2}=10\;\text{cm}$	10°	0.905	0.912	0.7	0.906	0.1
	15°	0.863	0.869	0.7	0.864	0.1
	20°	0.823	0.829	0.8	0.824	0.2
	25°	0.785	0.790	0.6	0.785	0.0
	30°	0.748	0.752	0.5	0.747	‐0.1
	45°	0.631	0.635	0.7	0.631	0.0
	60°	0.498	0.502	0.8	0.497	0.0

#### A.2. PDD

Considering that the collimator jaw is moving during the irradiation, depth doses and dose profiles can not be measured using the traditional tools (ionization chamber or silicon diodes coupled with a water phantom) in continuous scanning. Consequently, 98 single‐point integrated measurements were performed at several depths from $d_{\max}$ to 30 cm for field sizes of $10\times 10\;{\text{cm}}^{2}$ and $20\times 20\;{\text{cm}}^{2}$. The absorbed dose was measured for a fixed number of monitor units for each dynamic wedge angle. All measurements were normalized at 10 cm depth.

### B. off‐axis

#### B.1. Relative Dosimetry

Relative dose profiles were measured at 10 cm depth for 15°, 30°, 45° and 60° dynamic wedge angle for symmetric $10\times 10\;{\text{cm}}^{2}$ and $20\times 20\;{\text{cm}}^{2}$ fields, a half‐blocked $10\times 10\;{\text{cm}}^{2}$ field with $Y_{2}$ at 10 cm and $Y_{1}$ initially on the central axis, and a $10\times 10\;{\text{cm}}^{2}$ asymmetric field with $Y_{2}$ at 8 cm and the initial position of $Y_{1}$ jaw 2 cm away from the central axis.

#### B.2. Absolute Dosimetry

Absolute 2D dose distributions were measured at 10 cm depth for all the permitted dynamic wedge angles with symmetric $5\times 5\;{\text{cm}}^{2}$, $10\times 10\;{\text{cm}}^{2}$, $15\times 15\;{\text{cm}}^{2}$, and $20\times 20\;{\text{cm}}^{2}$ fields, as well as the two asymmetric fields illustrated before, for a total of 168 measurements.

## III. RESULTS

### A. Beam Axis

#### A.1. EDW Factors

In Tables 1 and 2, the values of any single EDW factor and the percentage difference between measured and calculated data are reported for both the energies and the calculation algorithms.

For the 6 MV photon beam, the average deviation (defined as the arithmetic mean of the absolute value of the percentage differences) is 0.4%±0.3% for both the algorithms. For the 15 MV photon beam, the average deviations are equal to 0.4%±0.3% and 0.2%±0.2% for PB and CC algorithm, respectively. In general, the agreement between measured and calculated data is within 1%, with a maximum discrepancy of up to 1.3% for the 6 MV photon beam, 60° wedge angle and $5\times 5\;{\text{cm}}^{2}$ field size.

#### A.2. PDD

Depth doses, as calculated by the treatment planning system, agree well with measured ones; the deviations for the seven wedge angles are never larger than 1% for both energies and algorithms.

### B. off‐axis

#### B.1. Relative Dosimetry

The comparison of the measured and calculated off‐axis profiles shows that a good agreement is achieved and no significant differences are observed between the algorithms.

**Figure 1:**
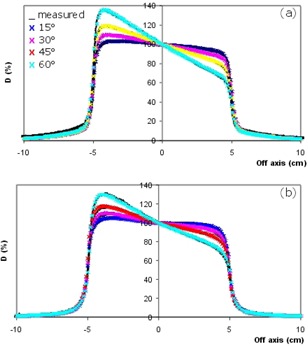
Dose profiles at different angles for a $10\times 10\;{\text{cm}}^{2}$ wedged field. Lines represent measured data; crosses represent calculated data obtained by using Pencil Beam algorithm at 6 MV (a) and 15 MV (b) photon energies, respectively.

**Figure 2:**
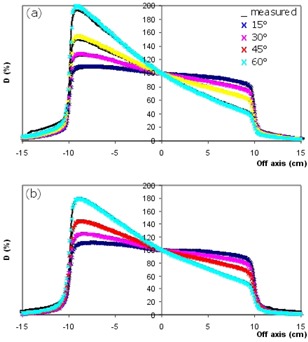
Dose profiles at different angles for a $20\times 20\;{\text{cm}}^{2}$ wedged field. Lines represent measured data; crosses represent calculated data obtained by using Collapsed Cone algorithm at 6 MV (a) and 15 MV (b) photon energies, respectively.

In the HDLG region, profiles for all field sizes and wedge angles are practically superimposed, as illustrated in Fig. 1 for the $10\times 10\;{\text{cm}}^{2}$. The largest deviations are less than 2% for both the energies. As illustrated in Fig. 2, on the high dose side (toe) of the profiles, better results are obtained for 15 MV beam energy, with maximum deviations below 2.5% for all the analyzed test cases. The lowest energy shows similar values for wedge angles up to 30 degrees EDW; the disagreement can increase to more than 3% for larger wedge angles using large field sizes.

Dose agreement in the HDHG region has been evaluated in terms of position errors. The differences between measured and calculated radiological field width are always less than 1 mm for all the tested cases.

In general, the agreement between measured and calculated profiles in the LDLG region is within the acceptance criterion of 3% recommended by Van Dyk et al.^(14)^ However, differences greater than this tolerance level are found for the largest field size and for wedge angles greater than 30 degrees (Fig. 2).

#### B.2. Absolute Dosimetry

The good agreement between measured and calculated data arising from the analysis of dose profiles is confirmed also by the comparison of 2D dose maps that, using acceptance criteria for DD and DTA equal to 3% and 3 mm respectively, shows a percentage of examined points resulting in γ values lower than or equal to 1 in more than 95% of all the tested cases, for both energies and calculation algorithms. Using more restrictive acceptance criteria (2%, 2 mm), the passing rate decreases; however, more than 90% of cases show the same good agreement between measured and calculated data.

**Figure 3:**
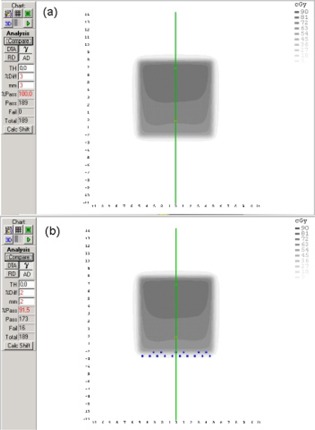
Measured and calculated (using Collapsed Cone algorithm) dose maps for 15 MV photon energy, $10\times 10\;{\text{cm}}^{2}$ asymmetric field size, 30° EDW angle. Data are for dose difference and distance‐to‐agreement acceptance criteria of 3%, 3 mm (a) and 2%, 2 mm (b), respectively.

For example, in Fig. 3 the results obtained for 15 MV, $10\times 10\;{\text{cm}}^{2}$ asymmetric field size, 30° EDW angle and CC algorithm are reported. In this case, the passing rate decreases from 100% to 91% after setting the acceptance criteria to 3%/3 mm and 2%/2 mm, respectively.

## IV. DISCUSSION

Results confirm the accuracy of Oncentra MasterPlan EDW calculations. EDW factors calculated by the TPS agree with measurements to within 1% over the range of field sizes that commonly occur in clinical practice. The 1% agreement is substantially aligned with the results obtained for other TPSs by Weber et al.^(4)^ for Helax, Papatheodorou et al.^(6)^ for iSiS3D, Miften et al.^(7)^ for Focus, Shao et al.^(8)^ for ADAC Pinnacle, and Caprile et al.^(10)^ for Varian Eclipse. It is evident then that the Oncentra MasterPlan TPS performs EDW output factors as well as the other commercially available TPSs.

EDW factor results are also consistent for comparable energies and fields within ±1% with those of Klein et al.,^(16)^ Gibbons^(17)^ and Prado et al.^(18)^ (Note: Data obtained by comparing our results with corresponding data extracted from EDW output factors curves or tables of the three cited papers.)

The analysis of depth dose values shows that Oncentra MasterPlan TPS achieves the same good results as reported by Weber et al.^(4)^ and Caprile et al.^(10)^ for Helax TMS and Varian Eclipse TPS, respectively.

For off‐axis dose distributions, calculated dose profiles agree well with the measured ones. In particular, the differences in the HDLG and HDHG regions are not only always less than the acceptance criteria of Van Dyk's^(14)^ analysis (3% and 4 mm, respectively), but are also always less than 2% and 2 mm, which represent the acceptance limits recommended by the ICRU^(19)^ in points of clinical relevance. These findings are consistent with those found by all other authors we reference in this paper. As did Chelminski et al.^(5)^, Shao et al.^(8)^ and Caprile et al.,^(10)^ for Helax, ADAC Pinnacle and Varian Eclipse TPS, respectively we found a slight disagreement in the edge of the toe for the lower energy. These results do not represent a serious limitation of the system, as they are limited to the last 5 mm of the toe.

In the LDLG region, the calculated profiles underestimate the measured ones; however, only when using large field size and wedge angle larger than 30 degrees. In these instances, the acceptance criteria of Van Dyk's^(14)^ analysis (maximum of 3% dose difference) are not fulfilled.

The good accuracy of the calculation algorithms observed in the comparison of off‐axis profiles in the wedged direction is extended to the plane perpendicular to the beam axis by the gamma analysis of the absolute 2D dose distributions calculated by the TPS and measured with the diode matrix.

It is important to note that both the 1D and 2D analysis do not show any significant differences between the calculation algorithms.

Finally, it should be stressed that the results demonstrate that Oncentra MasterPlan TPS can accurately model Varian EDW.

## V. CONCLUSIONS

The enhanced dynamic wedges have many advantages as compared with physical wedges, both for dosimetrical (beam hardening, peripheral doses) and for practical reasons. In order to implement the EDW in Oncentra MasterPlan TPS, the only input data are the SGTT files used in conjunction with the conventional output factors measured in air and water for open static beams. Therefore, the introduction of EDW in the clinical routine requires a correct analysis of the accuracy of the TPS in reproducing the wedged dose distributions.

The present work shows that the pencil beam and collapsed‐cone algorithms of the Oncentra MasterPlan TPS accurately model the wedged dose distributions created by the enhanced dynamic wedge of Varian's linear accelerator.

## ACKNOWLEDGEMENTS

The authors would like to thank Dr. Giuseppe Alberta, Nucletron (Veenendaal, The Netherlands), for providing all relevant information about the Oncentra MasterPlan EDW model.

## References

[1] Klein EE , Low DA , Meigooni AS , Purdy JA . Dosimetry and clinical implementation of dynamic wedge. Int J Radiat Oncol Biol Phys. 1995;31(3):583–92. 7852124 10.1016/0360-3016(94)00369-V

[2] Leavitt DD , Moeller JH , Stone A . Reduction of peripheral dose by dynamic wedge techniques. Med Phys. 1993;20:877.

[3] Ahlgren L , Olsson LE . Induced activity in a high‐energy linear accelerator. Phys Med Biol. 1988;33(3):351–4. 3362973 10.1088/0031-9155/33/3/004

[4] Weber L , Ahnesjö A , Nilsson P , Saxner M , Knöös T . Verification and implementation of dynamic wedge calculations in a treatment planning system based on a dose‐to‐energy‐fluence formalism. Med Phys. 1996;23(3):307–16. 8815372 10.1118/1.597797

[5] Chelminski K , Bulski W , Rostkowska J , Kania M . Dynamic wedges – dosimetry and quality control. Rep Pract Oncol Radiother. 2006;11(2):67–75.

[6] Papatheodorou S , Zefkili S , Rosenwald JC . The ‘equivalent wedge’ implementation of the Varian Enhanced Dynamic Wedge (EDW) into a treatment planning system. Phys Med Biol. 1999;44(2):509–24. 10070798 10.1088/0031-9155/44/2/016

[7] Miften M , Wiesmeyer M , Beavis A , Takahashi K , Broad S . Implementation of enhanced dynamic wedge in the focus rtp system. Med Dosim. 2000; 25(2):81–6. 10856686 10.1016/s0958-3947(00)00033-9

[8] Shao H , Wu X , Luo C , Crooks A , Bernstein A , Markoe A . The accuracy of dynamic wedge dose computation in the ADAC Pinnacle RTP system. J App Clin Med Phys. 2004;5(4):46–54. 10.1120/jacmp.v5i4.1964 PMC5723519 15738920

[9] Alaei P , Higgins PD , Gerbi BJ . Implemetation of enhanced dynamic wedges in Pinnacle treatment planning system. Med Dosim. 2005;30(4):228–232. 16275565 10.1016/j.meddos.2005.08.003

[10] Caprile PF , Venencia CD , Besa P . Comparison between measured and calculated dynamic wedge dose distributions using the anisotropic analytic algorithm and pencil‐beam convolution. J App Clin Med Phys. 2006;8(1):47–54. 10.1120/jacmp.v8i1.2370 PMC5722401 17592453

[11] Ahnesjö A , Saxner M , Trepp A . A pencil beam model for photon dose calculation. Med Phys. 1992;19(2):263–73. 1584117 10.1118/1.596856

[12] Ahnesjö A . Collapsed cone convolution of radiant energy for photon dose calculation in heterogeneous media. Med Phys. 1989;16(4):577–92. 2770632 10.1118/1.596360

[13] Low DA , Harms, WB , Mutic, S , Purdy, JA . A technique for the quantitative evaluation of dose distributions. Med Phys. 1998;25(5):656–61. 9608475 10.1118/1.598248

[14] Van Dyk J , Barnett RB , Cygler JE , Shragge PC . Commissioning and quality assurance of treatment planning computers. Int J Rad Onc Biol Phys. 1993;26(2):261–73. 10.1016/0360-3016(93)90206-b 8491684

[15] Buonamici FB , Compagnucci A , Marrazzo L , Russo S , Bucciolini M . An intercomparison between film dosimetry and diode matrix for IMRT quality assurance. Med Phys. 2007;34(4):1372–79. 17500468 10.1118/1.2713426

[16] Klein EE , Gerber R , Zhu XR , Oehmke F , Purdy JA . Multiple machine implementation of enhanced dynamic wedge. Int J Rad Onc Biol Phys. 1998;40(4):977–85. 10.1016/s0360-3016(97)00916-4 9531384

[17] Gibbons JP . Calculation of enhanced dynamic wedge factors for symmetric and asymmetric photon fields. Med Phys. 1998;25(8):1411–18. 9725127 10.1118/1.598313

[18] Prado KL , Kirsner SM , Kudchadker RJ , Steadham RE , Lane RG . Enhanced dynamic wedge factors at off‐axis points in asymmetric fields. J App Clin Med Phys. 2003;4(1):75–84. 10.1120/jacmp.v4i1.2544 PMC5724440 12540821

[19] International Commission of Radiation Units and Measurements . Use of computers in external beam radiotherapy procedures with high energy photons and electrons. ICRU Report 42. Bethesda (MD): ICRU Publications; 1987.

